# An Alternative to the Orthodoxy in Animal Ethics? Limits and Merits of the Wittgensteinian Critique of Moral Individualism

**DOI:** 10.3390/ani9121057

**Published:** 2019-12-01

**Authors:** Susana Monsó, Herwig Grimm

**Affiliations:** Unit of Ethics and Human-Animal-Studies, Messerli Research Institute, University of Veterinary Medicine, Vienna, Medical University of Vienna, University of Vienna, 1210 Vienna, Austria; herwig.grimm@vetmeduni.ac.at

**Keywords:** animal ethics, moral individualism, Alice Crary, Cora Diamond, utilitarianism, axiology

## Abstract

**Simple Summary:**

Traditional approaches to animal ethics depart from the assumption that the moral consideration that we owe to different beings depends on their individual characteristics—that if we want to know how to treat a particular animal, the answer is ultimately “in the animal”. This view, which has come to be known as “moral individualism”, has been criticised by the Wittgensteinian authors Cora Diamond and Alice Crary. In this paper, we argue that there are severe limitations to this criticism, which make the target of their critique significantly smaller than these authors presume. At the same time, we also argue that there are important merits to their critique, and that we should incorporate several of their insights into our reflections on how to treat other animals.

**Abstract:**

In this paper, we analyse the Wittgensteinian critique of the orthodoxy in animal ethics that has been championed by Cora Diamond and Alice Crary. While Crary frames it as a critique of “moral individualism”, we show that their criticism applies most prominently to certain *forms* of moral individualism (namely, those that follow hedonistic or preference-satisfaction axiologies), and not to moral individualism in itself. Indeed, there is a concrete sense in which the moral individualistic stance cannot be escaped, and we believe that it is this particular limitation that justified Crary’s later move to a qualified version of moral individualism. At the same time, we also argue that there are significant merits to the Wittgensteinian critique of moral individualism, which pertain to its attack on the rationalism, naturalism, and reductionism that characterise orthodox approaches to animal ethics. We show that there is much of value in the Wittgensteinians’ call for an ethics that is more *human*; an ethics that fully embraces the capacities we are endowed with and one that pays heed to the richness and complexity of our moral lives.

## 1. Introduction

In 2010, Alice Crary published a paper entitled “Minding what already matters: A critique of moral individualism” [1]. In this paper, she criticised the theoretical approaches to animal ethics that were predominant at the time, and which can still be considered as the orthodoxy. These are the approaches paradigmatically championed by Peter Singer and Tom Regan, which despite their significant differences Crary grouped under the label of “moral individualism”. Moral individualism (hereafter, “MI”), in rough terms, is the view that the moral consideration that we owe to different beings depends on their individual characteristics—that if we want to know how to treat a particular animal, the answer is ultimately “in the animal”. Crary levelled a heavy critique against MI, accusing it of both departing from “wrongheaded assumptions about the nature of our moral relationship to other human beings” and of leading us to “distorted images of our moral relationships with animals” [1] (p. 20). Some years later, however, Crary seemed to partially retract her original criticism, apparently defending that MI was not to be completely abandoned, but rather that, instead of adopting “traditional MI”, we should go for “alternative MI” [2] (p. 164). In this paper, we want to assess the limits and merits of her original critique of MI, and, in the process, we reveal the reasons that might have prompted Crary to this modification of her view.

Crary, together with Cora Diamond, represent the Wittgensteinian approach to animal ethics^1^, which at its core is significantly contrary to the tenets of moral individualists such as Singer and Regan. We want to show that there are important limitations to the critique of MI originally defended by these Wittgensteinian authors—their criticism applies most prominently to certain *forms* of MI, but not to MI in itself. Moreover, there is a concrete sense in which MI cannot be escaped, and we believe that it is this particular limitation that justified Crary’s move to a qualified version of MI. However, we also want to show that in certain respects the original criticism still stands, and indeed that there are several important lessons to be learnt from it. Accordingly, our paper follows the following structure. In Section 2, we begin by sketching the Wittgensteinian critique of MI. In Section 3, we delineate its limits. In Section 4, we highlight its merits. Lastly, in Section 5, we respond to one of the most common objections against the Wittgenstenian account and clarify to what extent this approach amounts to a full-blown alternative to the orthodoxy. Our aim in this paper is not to provide a defence of the theories of Crary and Diamond,^2^ but instead to provide an analysis of the strengths and weaknesses of their critique of the orthodoxy, which can ultimately contribute to clarifying which aspects of it should be preserved, and which might have to be modified or altogether relinquished.

As a final clarification, throughout the text, we use the term “orthodoxy” to refer mainly to the seminal texts in animal ethics, namely *Animal Liberation* [4] and *The Case for Animal Rights* [5], but also to the works of those authors who utilise similar theoretical frameworks, such as those by DeGrazia [6], Francione [7], McMahan [8], Pluhar [9], Rollin [10] and Varner [11]. Although Crary uses the term “MI” as practically synonymous with the orthodoxy, we want to differentiate these two terms. MI, we argue, is a meta-ethical principle that is followed by the orthodox authors and many other ethical theories beyond, thus it is imprecise to equate MI with the orthodoxy. Instead, for reasons that are made clear below, we argue that the term “traditional MI” should be preferable to refer to the orthodoxy.

## 2. The Wittgensteinian Critique of MI

Crary’s [1] critique of MI is a development of the ideas already formulated by Diamond in her critique of orthodox approaches to animal ethics, first defended in her 1978 paper “Eating meat and eating people” [12]. Though Diamond does not phrase her ideas as a criticism of MI in these terms, the features that Crary will identify as central to MI constitute a clear focus of Diamond’s original critique. Therefore, while our analysis primarily refers to Crary, we often draw on Diamond as a way of illustrating the core ideas that Crary developed in her critique of MI. Crary and Diamond also share the fact that they both adhere to Wittgensteinian ideas in their ethics and metaphysics, and indeed Wittgenstein’s thinking underlies both their critique of the orthodox approach to animal ethics and the alternative account of morality that they offer instead. While, as De Mesel and Thompson point out, it is difficult to delineate Wittgenstein’s ethical views, commentators tend to agree that “he eschewed reductionism and scientism in moral philosophy as elsewhere, and that he did not offer normative guidance in form of a decisional procedure that could help us to solve moral problems or to decide what to do in particular cases” [13] (p. 1).^3^ Due to space constraints, in this paper, we mostly focus on the negative side of Wittgensteinian thinking, in the form of the critique of the orthodoxy developed by Diamond and Crary, leaving a thorough discussion of the positive account of animal ethics derived from Wittgenstein for future work.

Diamond and Crary criticise the orthodoxy in animal ethics for the widely held view that merely being human is not of ethical relevance. The most popular argument in animal ethics, which was already formulated in *Animal Liberation* in 1975 [4] and has since been echoed countless times in a myriad of forms, precisely departs from the idea that merely being human is not of ethical relevance. This is the argument from “marginal cases”, also known as the argument from species overlap [21]. According to this argument, there is no single capacity that all humans share and no other animal has that could serve to justify giving preferential treatment to humans. The most commonly cited capacities that supposedly set us apart from animals (language, rationality, moral agency, etc.) are in fact not shared by all human beings. Therefore, they cannot be used to ground a preferential treatment for *all* members of the human species. If we pick a less cognitively demanding capacity as grounds for ethical treatment, such as sentience, then many animals will also fall within its scope. Thus, giving preferential treatment to humans cannot be rationally grounded.^4^ However, the argument only works if one assumes that the one thing that *is* shared by all humans, namely, their belonging to the human species, cannot be used as grounds for preferential treatment. This is usually assumed to be the case, since merely being human is deemed morally irrelevant. Accordingly, the argument from “marginal cases” is usually accompanied by an appeal to the need to avoid speciesism [23]: an illegitimate bias in favour of members of one’s own species merely on the grounds that they are members of one’s own species. Speciesism is construed as analogous to racism or sexism, and it is considered a vice that should be avoided at all costs.

Crary and Diamond are both highly critical of this argument, not because they disagree with the need to protect animals from unethical treatment, but because they believe that this argument both distorts our understanding of moral relationships with other humans—insofar as these are not dependent on cold calculi regarding their possession or otherwise of the requisite capacities—and gives uncompelling grounds for the ethical treatment of animals. The argument from “marginal cases”, in their view, has the repugnant implication that one could simply respond to it by worsening the treatment of “marginal” humans rather than improving the treatment of animals. Moreover, the Wittgensteinians argue that the orthodoxy in animal ethics simply cannot give a proper account of our lived morality. The way in which we care for disabled people, for instance, illustrates how “diminished” capacities are not generally viewed as grounds for worse treatment.

The core claim of moral individualism asks us to regard the impairments of the cognitively radically impaired as drastically weakening their claim to moral consideration. The claim thus flies in the face of the thought that, precisely in view of their special susceptibility, cognitively impaired human beings merit special solicitude.

[1] (p. 21)

Moreover, they argue that the orthodoxy ignores how human societies treat *dead* humans. Here, there is not just an absence of cognitively demanding capacities, but an absence of all form of subjectivity. However, we treat dead human bodies in respectful ways: we believe that they are not things to be defaced, mutilated or displayed in a degrading manner, let alone eaten. The argument from marginal cases, they argue, cannot even begin to explain why we tend to consider that a cow that is struck by lightning could be eaten while it would be unthinkable if the unlucky individual were a human [12] (p. 468).

For the authors in the orthodox tradition, our refusal to eat human corpses amounts to little more than a cultural quirk, while Diamond and Crary consider it as one of those practices that precisely helps us to understand what being human *consists of.* Diamond writes:

If the point of the Singer–Regan vegetarian’s argument is to show that the eating of meat is, morally, in the same position as the eating of human flesh, he is not consistent unless he says that it is just squeamishness, or something like that, which stops us eating our dead. If he admitted that what underlies our attitude to dining on ourselves is the view that *a person is not something to eat*, he could not focus on the cow’s right not to be killed or maltreated, as if that were the heart of it.

[12] (p. 468)

It is not simply “morally wrong” to eat a human being, nor is it something that we refuse to do out of respect for people’s interests; it is instead precisely one of those practices that “go to determine what sort of concept ‘human being’ is” [12] (p. 470). Being human *means* being something that is not to be eaten.^5^ By ignoring this fact, what Singer and Regan are doing “is not to give a defence of animals; it is to attack significance in human life” [12] (p. 471). For the Wittgensteinians, defending animals is not a matter of erasing the significance in human life, but of adding significance to how we speak and think of animals and consequently how we understand the world and ourselves. This is not to be done by uncovering biological facts about their capacities, but it is instead a task for inquiries into the use of language, artistic endeavours, and narratives that employ our moral imagination. In terms of methodology, this implies, for instance, the use of literature to expand moral imagination and significance, rather than the search for morally relevant biological facts. 

Both Crary and Diamond identify the tendency of orthodox authors to approach the world as though it were devoid of values as the crucial factor behind the unappealing portrayal of morality they develop. This is precisely what Crary identifies with MI. MI, as a principle that grounds the ethical treatment of others in their individual characteristics, inclines us to look at the world as “objectively” as possible. Since facts are viewed as the grounds for morality, it is crucial to get them straight, which brings MI close to the scientific world view, mirrored in the idea that there is a spectator-neutral world that can be described objectively.^6^ At the same time, it is assumed that the world “is neutral in the sense of being given to ethics independently of the exercise of moral capacities” [1] (p. 41). The problem that both Crary and Diamond see is that the concepts of “human” and “animal” are not merely biological. There is a sense in which they refer to beings with certain biological characteristics, but if we only think about the biological sense of these concepts, we are also leaving much aside, much that is genuinely there and important for our lived morality. In Crary’s words, “when we talk about human beings and animals in ethics we are talking about beings such that moral reflection is required in order to bring them clearly into focus” [1] (p. 41). By adopting an approach that only recognises the biological sense of these concepts, proponents of MI are offering a partial and distorted account of morality and our moral life.

While we believe that there are several elements to be praised in this critique of the orthodoxy in animal ethics, we also think that the criticism is to a certain extent clouded by its incorporation of some argumentative flaws and conceptual confusions. Having given this quick sketch of the Wittgensteinian critique of MI, we now turn to delineating its limits (Section 3) and subsequently defend its merits (Section 4). This will reveal the sense in which MI should be preserved, and the sense in which it might have to be relinquished. 

## 3. Limits

A good starting point to delineating the limitations of the Wittgensteinian critique of MI is to go back to the first explicit definition that was given of MI. This was formulated by James Rachels, and it goes as follows: “[t]he basic idea is that how an individual may be treated is to be determined, not by considering his group memberships, but by considering his own particular characteristics” [24] (p. 173). There are two significant differences between this original definition and how Crary later characterised MI, and these two differences make Crary’s notion of MI significantly narrower, thus in principle reducing the amount of theories that her notion applies to. Crary writes:

What distinguishes approaches in ethics that count as forms of *moral individualism* is the claim that a human or nonhuman creature calls for specific forms of treatment only insofar as it has individual capacities such as, for instance, the capacity for suffering or the capacity to direct its own life.

[1] (p. 20, emphasis in the original)

The first difference between Rachels’ original definition and Crary’s reformulation is that, where Rachels speaks of *characteristics*, Crary speaks of *capacities*. This is not an insignificant change^7^ as the notion of a characteristic is significantly broader than that of a capacity. All the capacities of an individual are also characteristics of hers, but not vice versa, so a characteristic-based MI would prima facie encompass more theories than a capacity-based MI. 

Crary’s reduction of MI to a capacity-based form of it is not an isolated event in animal meta-ethics. It is also present, for instance, in May’s distinction between MI and moral relationalism. May defines moral relationalism as “the view that the moral status of a living being is determined not by solely its particular characteristics but more importantly by the relations it has with human beings” [25] (p.155). He contrasts moral relationalism with MI, which he considers to take capacity-based reasons as grounds for moral standing, rather than relation-based reasons. This distinction appears to be misguided, for the simple fact that relations are also characteristics of individuals, and so it is unclear why moral relationalism should not be viewed as a form of MI. Likewise, Delon [26] identifies MI with the view that “the only properties of entities that matter to their moral status are *intrinsic* properties (typically psychological or cognitive capacities such as sentience or consciousness)” (p. 31), a view he contrasts with theories that also take into account *extrinsic* properties, such as the surrounding context or pre-existing relations. The view that Delon calls MI would more aptly be labelled “moral intrinsicalism”,^8^ since extrinsic properties are also characteristics that belong to individuals, even if they are not intrinsic to them. If we depart from Rachels’ original definition, then, moral relationalism would be a form of MI that particularly emphasises the group of characteristics that fall under the umbrella of “relations towards humans”, and moral intrinsicalism would be a form of MI that highlights the group of characteristics that can be considered “intrinsic properties”. Insofar as both capacities and relations are characteristics of individuals, the sort of MI originally formulated by Rachels can be viewed as encompassing both approaches that feature intrinsic, capacity-based reasoning (e.g., Singer’s utilitarianism, Regan’s rights theory, or Nussbaum’s capabilities approach [27]) and those that, in addition, emphasise extrinsic, relation-based reasoning (e.g., Palmer’s contextual approach [28]).^9^

We believe that Crary’s conflation of MI with a capacity-based MI is at the heart of most of the problems we identify in her critique. We return to this below, but first we want to point out the second difference between Rachels’ and Crary’s definition of MI. In Rachels’ original formulation, MI is considered a metaethical principle governing how to justify differences in treatment across individuals. The idea is that one cannot legitimately treat two individuals differently without being able to cite a morally relevant difference between the two individuals. It is thus meant as a principle that guarantees the equal consideration of equals. Crary, however, makes MI a claim about *moral status*. For moral individualists, according to her account, whether and to what extent an entity has moral status is a function of her individual capacities [2] (p. 158). While this is not so clear in her initial definition, it appears time and again in her writings. For instance, she asserts that moral individualists hold “that only intrinsic characteristics endow a creature with ‘moral status’ in virtue of which it is a source of agent-neutral reasons” [1] (p. 21), that “[m]oral individualists claim that it is only insofar as individual animals have certain mental capacities that they merit moral consideration” [1] (p. 41), and that MI is a “doctrine that grounds moral status in individual capacities” [2] (p. 158). By making MI a doctrine about moral status in particular, rather than a principle governing ethical judgements more generally, Crary is also narrowing the amount of theories that can be considered forms of MI, insofar as some theories that do not incorporate the notion of moral status, such as virtue ethics [29], are excluded from counting as MI. However, it is doubtful that virtue ethicists would not subscribe to the equal consideration of equals, more so given the Aristotelian origins of this principle.

This is not to say, however, that Rachels’ original definition should be accepted without any critical examination. In fact, by looking closely at it we can identify at least one problem. Rachels, as mentioned above, characterises MI as the idea that “how an individual may be treated is to be determined, not by considering his group memberships, but by considering his own particular characteristics” [24] (p. 173). There is something that immediately strikes the eye here, namely, that one’s group memberships are also part of one’s characteristics. A way out of this apparent contradiction might be to claim that MI excludes group memberships from the characteristics that can determine how an individual ought to be treated. However, this would turn MI into an absurd principle, for there undoubtedly exist *some* group memberships that legitimately determine how an individual ought to be treated. For instance, imagine that the coach of a football team decides to forbid anyone who is not a member of the team from entering the field during training sessions. He would clearly be treating individuals differently based on their group membership, but this does not appear to be a violation of the equality principle. It would be absurd for a random person who is not a member of the team to claim she is being discriminated or treated unjustly by not being allowed onto the field, and it is absurd precisely *because* she does not belong to the group “football team”. Even if she is in perfect physical conditions and an excellent football player, it is still completely legitimate to exclude her from the training session for the precise reason that she is not part of the team that is training there. In this sense, being a member of a group can sometimes turn equals into unequals, thus legitimately grounding a difference in treatment. Therefore, group memberships—even though they can be considered extrinsic, rather than intrinsic properties—cannot be excluded tout court from the list of characteristics that determine how an individual ought to be treated.

It seems, however, that what Rachels had in mind when he spoke of excluding group memberships was not group memberships in general, but *species* membership in particular [24] (p. 173f.). Indeed, he immediately clarifies that MI is meant to secure that differences in treatment “cannot be justified by pointing out that one or the other is a member of some preferred group, not even the ‘group’ of human beings” [24] (p. 174). This definition of MI is thus tailored to exclude speciesism: a difference in treatment based solely on a difference in species membership. However, the reason why speciesism has since its original formulation been considered a form of discrimination is *not* because belonging to a species amounts to belonging to a *group*, but because species membership is generally thought to be a *morally irrelevant characteristic* of individuals. If two individuals are to be treated differently in the same circumstances, one should be able to justify this by pointing to a characteristic that is relevant to the difference in treatment. This idea is mirrored in Rachels’ formulation of the principle of equality: “Individuals are to be treated in the same way unless there is a *relevant* difference between them that justifies a difference in treatment” [24] (p. 176, our emphasis). Although within some worldviews being human translates into a morally relevant characteristic, because it amounts, for instance, to having been created in the image of God, within contemporary philosophy, it has traditionally been considered that species membership cannot function as a relevant difference in this sense.

Despite having often been invoked against speciesism, the principle of equality thus formulated, as with MI itself, is in fact neutral with respect to both the normative framework and the axiology one should follow. Neither principle specifies *how* individuals ought to be treated nor *what counts* as a morally relevant difference. This is why a rejection of speciesism does not automatically follow from the principle of equality, and also why Crary’s critique of MI for denying the moral relevance of being human is, at heart, misguided. Being human is still a *characteristic* that applies to *individuals*, so a theory that derives entitlements from being human would nevertheless count as MI. While moral individualists traditionally consider being human a *morally irrelevant* characteristic (because they conceive of it solely in biological terms), this is not a *necessary* trait of MI, but rather depends on one’s criteria of moral relevance. MI is for instance compatible with claiming that being human is a morally relevant characteristic because it implies having been created in the image of God, or because only humans can participate in person-rearing relationships [30], or because only humans have a root-capacity for morality [31].

In fact, Crary and Diamond’s arguments amount to a defence of the idea that being human is morally relevant, because they understand moral relevance as what is *de facto*, in a descriptive sense, morally relevant.^10^ The Wittgensteinian critique is thus not directed at the very idea of MI, but at the traditional exclusion by moral individualists of being human as a morally relevant characteristic. In Crary’s and Diamond’s view, this does not correspond to our lived morality, which is why they consider it a fundamental flaw in orthodox approaches to animal ethics. However, by noting that being human is also a characteristic of individuals, the Wittgensteinians’ claim that merely being human is morally significant becomes perfectly compatible with MI.

Crary and Diamond not only have qualms about the orthodoxy’s traditional exclusion of being human from the set of morally relevant characteristics, they also take issue with moral individualists’ tendency to reduce the morally relevant characteristics of individuals to a single capacity or set of capacities. As we have seen, the Wittgensteinians argue that this has the objectionable implication that severely disabled or dead humans merit less or no moral consideration, due to the fact that they have reduced or absent capacities.^11^ We do not want to deny that this follows from *some* forms of MI, such as Singer or McMahan’s utilitarianism, but we want to stress that this is a *contingent* characteristic of certain forms of MI that follows from their particular axiologies. A normative theory that departs from an axiology that only recognises *subjectively experienced* forms of harm, such as hedonism, cannot account for the ways in which dead bodies may be disrespected or forms of harm that affect disabled people when they cannot register them as such. We think that this is a severe flaw of hedonistic and preference utilitarianism.^12^ This is also true of other theories with similar axiologies, but this flaw stems *from their axiology*, and *not* from their moral individualistic character.^13^

In fact, MI is in principle perfectly compatible with an axiology that acknowledges objective values and objective harm, for, as already pointed out, MI is an axiologically-neutral principle. Moreover, Crary and Diamond themselves seem to take an individualistic approach to justifying our duties towards disabled and dead humans, for they ground these duties in an appeal to the *individual characteristics* of these entities. For instance, Diamond writes:

It would be held by many people uninfluenced by theories of what can count as moral relevance that the conviction by a court of a severely retarded [*sic*] person for a crime that required an intention the retarded person could not form was unjust; the less capable of forming such an intention the person is, the more palpable the injustice.

[33] (p. 53)

If a person with a severe cognitive disability is convicted of a crime that requires an intention she could not have formed, it is precisely the fact that she has these *individual characteristics* that make the injustice so blatant. This is an individualistic account of the harm that befalls this person. Likewise, MI is perfectly compatible with the sort of conceptualisation of severely disabled humans that Crary endorses, namely, “as having suffered from a great deprivation in virtue of which they merit special solicitude” [1] (p. 33). This is a description that attends to the individual’s particular characteristics, and which can be construed as giving rise to special entitlements. In a similar vein, MI can serve to give an account of why someone who “takes advantage of a severely demented Alzheimer’s patient does something *particularly vile*” [1] (p. 22, our emphasis). It is particularly vile *because* of the individual’s particular characteristics, because of the increase in vulnerability that goes along with the disease’s effects. MI can also be used to explain the treatment owed to dead humans. Here, there is no subjectivity involved, so subjective harms are ruled out, but it is still possible to describe their bodies as “those of our fellows who have reached the end of their mortal threads” [1] (p. 34); an individualistic description that can, depending on our normative theory, serve to justify specific forms of respect that go beyond maximising pleasure or calculating preferences.

MI can therefore account for the existence of duties towards dead and disabled humans just as well as it can account for duties towards the living and the abled, so long as we are willing to depart from hedonistic and preference-satisfaction axiologies. However, there is a further sense in which the Wittgensteinian critique of MI misfires, and this is the fact that *some* form of individualism is ultimately inescapable. Regardless of the normative framework and axiology we depart from, regardless of how we construe moral psychology and of our underlying metaphysics, if we are doing ethics, it is hard to think how we could escape having to pay *some* attention to the individual we are dealing with and might suffer from our actions. We might be willing to construe species membership as morally relevant or not, we might think that moral status is a useful notion or not, but *what characterises* the individual we are dealing with is always going to be a relevant piece of information, if only to determine whether it is a living being or an inanimate entity, whether it is an artefact, a dead body, and so on. One can see easily see this by looking beyond utilitarian and rights approaches to animal ethics. In virtue ethics, for instance, the features of the concrete situation one is in, including what characterises the individual is dealing with (e.g., “what species does she belong to?”, “what situation is she currently in?”, and “what is her relation to me?”), are fundamental to determining what the virtuous thing to do would be (see, e.g., Hursthouse [37]). Nussbaum’s capabilities approach also implies paying attention to the individual: determining what sort of thing an individual is will be crucial to establishing what constitutes flourishing for her and what sort of harms she can suffer (see, e.g., Nussbaum [27]). Even Palmer’s contextual approach [28], which famously emphasises contextual, historical and relational factors, cannot and indeed does not aim to escape paying attention to the individual: the causal origins of the vulnerabilities that characterise this particular individual are crucial in determining the sort of moral obligations that we have towards her.

The ultimate inescapability of MI, we think, is what lies at the heart of Crary’s partial retraction of her original critique. In her most recent paper on the topic, she writes, in reference to her preferred approaches to animal ethics:

[T]here are other thinkers who take up questions of animals and ethics and who, while differing substantially from Singer et al. in their views, nevertheless resemble them in basing their conclusions about moral standing on attention to individual creatures. In light of this convergence, it makes sense to withhold the generic label “moral individualism” from the projects of Singer and likeminded others and to place their work instead under the heading of *traditional moral individualism*.

[2] (p. 158)

In defending this alternative approach to animal ethics, Crary writes of the case, recounted in Gruen [38], of a chimp who was raised in the entertainment industry and trained to pull a ridiculous and presumably funny face when in the presence of humans. Nowadays, even though the chimp lives safely in a sanctuary, he still pulls that same face whenever he meets a human, automatically and unknowingly making a spectacle of himself. In being conditioned to behave in a way that Gruen regards as undignifying for the chimpanzee, this individual, Crary argues, was harmed in a sense that utilitarianism cannot capture, because the chimp himself is not able to register this harm. To capture this harm, however, we still have to pay “attention to him as an individual” [2] (p. 164). The fact that he is a chimpanzee, a majestic creature who in the wild we would be terrified to encounter, and the fact that he is *this* chimpanzee, with his particular history, makes us view the remnants of the training he received as a violation of his dignity. Crary, in the end, acknowledges that some attention to the individual is inescapable, and where before she spoke of the need to fully relinquish MI, Crary now speaks of a need for “nontraditional or alternative moral individualism” [2] (p. 164).

In this section, we show that there are severe limitations to the Wittgensteinian critique of MI. These limitations stem from the fact that the critique is not levelled at MI *in itself*, but at the way in which MI is applied in the orthodox accounts of animal ethics, and especially at the axiologies that traditionally accompany it. Moreover, we argue that there is a sense in which MI cannot be escaped, which is presumably what prompted Crary to move from a critique of MI tout court, to a critique of *traditional* MI. In the following section, however, we focus on the merits of the Wittgensteinian critique of MI. Even if MI ultimately cannot be escaped, there are certain facts about how orthodox forms of MI have developed, or about how MI has traditionally been interpreted, that can be brought into question. It is here, we argue, where the Wittgensteinian critique has the most to offer.

## 4. Merits

The problems associated with what Crary calls “traditional MI” were already identified by Diamond in her 1978 paper “Eating meat and eating people”. These problems stem not so much from the fact that the orthodox theories are *individualistic*, but from their possession of three further traits: (1) rationalism; (2) naturalism; and (3) reductionism. Although these three traits are not a necessary characteristic of MI, they do follow from an excessive focus on aseptically deriving ethical entitlements from the possession of certain characteristics. Moreover, to a certain extent, the three traits are interrelated and interdependent. In what follows, we explain these traits and evaluate the associated Wittgensteinian critique.

(1) Rationalism: Traditional MI is *rationalistic* in the sense that emotions are purposefully left aside in the discussion of what is owed to animals. Ethical theory is construed as a close-to-scientific endeavour, where anything that might cloud or distort rational discussion is shunned. In a famous passage of *Animal Liberation*, Singer describes how he is not particularly fond of animals, and that he was not moved by any sentiment of love towards animals to write that book. He explains that throughout the book he “makes no sentimental appeals for sympathy” towards animals, and that this work is instead “an attempt to think through, carefully and consistently, the question of how we ought to treat nonhuman animals” [4] (pp. xi, xii). While he acknowledges that the descriptions he makes of factory farming and animal experimentation will stir some emotions in the reader, the justification he gives for opposing these practices is “not emotional”, but instead “an appeal to basic moral principles which we all accept” where “the application of these principles...is demanded by reason, not emotion” [4] (p. xii). This attempt to leave all emotions aside and develop a defence of animal ethics in the most rational and aseptic way possible was echoed by Regan (see e.g., [5], (p. lii)) and has since been incorporated by most animal ethicists of the analytic tradition. It mirrors the idea that ethics is about creating rational consistency only (see e.g., [8,39]) and that immoral behaviour can be translated into irrational, i.e., inconsistent behaviour.
  The rationalist trend in the orthodoxy has been severely criticised by feminist writers, who have argued that emotions such as care are fundamental to treating animals how they should be treated (e.g., [40,41]). Several philosophers outside of the feminist tradition have also defended the importance of emotions in animal ethics (e.g., [42,43,44]). For the Wittgensteinians, especially Diamond, emotions such as pity are crucial for seeing animals in an ethical light. Pity, in her understanding, is fundamentally involved in our conception of suffering and death, in our coming to grasp both what they mean and why they matter to those who experience them, including animals. By making abstract appeals to the prevention of suffering, Singer and company are assuming that we can come to understand why suffering should be prevented without the involvement of emotions such as pity [12] (p. 478). Diamond sees this as confused, and she accuses the “philosophical over-emphasis on principles” that follows from this cold rationalism of alienating people from philosophy and making these philosophers’ arguments ultimately uncompelling [33] (p. 57).
  Diamond does not give much by way of arguments to defend the need to recruit emotions in animal ethics. However, even a cursory glance at the empirical evidence being gathered in the field of moral psychology supports the need to counter excessive rationalism. Already from Damasio’s work (e.g., [45]) we know that emotions are needed to move from moral judgement to action. However, accumulating evidence shows that emotions can influence and even determine moral judgements (see, e.g., [46]). Prinz, for instance, has argued with reference to empirical evidence that emotions and moral judgements are interrelated to the extent that emotions typically co-occur with moral judgements, emotions often influence moral judgements, and emotions are needed for proper moral development [47]. He mentions the case of psychopathy, where severe emotional deficits lead to an absence of empathic distress, remorse and guilt. Prinz even suggests that emotions may be necessary for moral judgements in a synchronic sense, to the extent that being committed to a moral judgement entails being disposed to feeling certain emotions in certain contexts. Regardless of how strongly one wants to commit to a sentimentalist account of morality, it is undoubtedly the case that emotions are involved to a certain extent in our moral lives. By leaving aside all emotions in one’s theory of animal ethics, one is failing to recruit this powerful ally, while simultaneously treating one’s interlocutors as purely rational—in the sense of non-emotional—and not as the highly emotional moral beings that we are.
(2) Naturalism: Traditional MI is *naturalistic* in the sense that it departs from empirical facts about animals and attempts to ground the reasons for the ethical treatment of animals in these facts. For instance, Singer’s utilitarian account of animal ethics departs from the biological fact that certain animals have certain capacities to experience pleasure and pain, and these capacities give rise to certain interests that are to be respected. It is assumed that if we know how the world *is*, we will be able to easily arrive at reasons for action, since these are ultimately grounded in natural facts; for instance, the fact that pain *feels* bad and generates an interest in not feeling pain implies that we should aim to minimise the amount of pain in the world. For the Wittgensteinians, this naturalism is problematic for two main reasons. The first reason is that it assumes that we can adopt the “point of view of the Universe” in our thinking about animals and what matters to them (see, e.g., [48]). This is connected to traditional moral individualists’ search for purely *rational* grounds for treating all beings ethically as impartial spectators. Attempting to adopt the point of view of the Universe is, in Diamond’s words, to attempt to find “reasons which are reasons for anyone, no matter how devoid of all human imagination and sympathy” [12] (p. 479). For Crary and Diamond, not only will this result in a moral theory that is uncompelling, thinking that we could adopt such an impartial and detached perspective is also little more than wishful thinking. They depart from what could be called an “epistemic anthropocentrism”: the view that we simply cannot escape our being human in our way of approaching and understanding the world. This, which proponents of traditional MI would regard as a regrettable loss of objectivity, is constructed by the Wittgensteinians as something that should be embraced and made the most of (see also [16], pp. 91–110). Instead of attempting to approach the world in the cold and aseptic manner of the scientist, the ethicist should make full use of the capacities involved in our moral lives, such as our moral imagination and our capacity to be moved by narratives.
  The second reason Crary and Diamond reject the naturalism of traditional MI is that it assumes that the grounds for morality are to be found in the characteristics of individuals, whereas the Wittgensteinians defend that the grounds for morality are provided by our practices, and that these practices also often inform how we conceptualise the characteristics of beings we interact with. As we have seen, the Wittgensteinians are not completely against paying attention to the *individual* one is dealing with, but our practices are understood to be very often at the root of how we conceptualise this individual (what characteristics we attribute to it) and also ultimately at the grounds of any justification for treating the individual in one way or another. In Diamond’s words:
  We can most naturally speak of a kind of action as morally wrong when we have some firm grasp of what *kind* of beings are involved. But there are some actions, like giving people names, that are part of the way we come to understand and indicate our recognition of *what* kind it is with which we are concerned.
  [12] (p. 469)
  We believe that this appeal to the importance of practices in the grounding of morality is one of the most insightful contributions of these Wittgensteinian authors, and one that the authors in the orthodoxy are apparently oblivious to, even though it is implicit in their argumentations. Indeed, the argument from “marginal cases”—as a paradigmatic case of orthodox argumentation—would not work at all were it not for a background of practices that support it. Imagine someone who tried to justify slaughtering and eating cows by pointing out that cows do not have a concept of death and so do not care about being killed and eaten so long as we do it painlessly. A possible response, using the argument from “marginal cases”, would be to claim that human babies do not have a concept of death either, and yet we do not eat them. If this argument does anything more than simply prove that there is an inconsistency in our interlocutor’s behaviour, this is because of the moral force that comes from our established practices, from the way in which we treat human babies despite their lack of language and concepts. If it is at all convincing, it is going to be because we manage to get our interlocutor to see the cow in a new light with help from the practices we established in the human realm.
  Despite this critique of the naturalism involved in traditional MI, it is important to note that it does not follow from Crary’s and Diamond’s work that all biological facts must be left aside. Knowing the biological facts pertaining to a particular animal is important, and in some cases crucial, for treating her properly. This is acknowledged by Diamond, for instance, when considering the concept of “friendship” in relation to animals. She claims she used to think friendship with animals “was obviously possible only in some cases, titmice and not hippopotamuses, e.g., but recent films of the relation between whales and their Greenpeace rescuers” made her realise that she “was probably taking an excessively narrow view” [12] (p. 475). Seeing whales in new light is made possible by learning of this practice of the Greenpeace rescuers, but it is also dependent upon biological facts of the whales (which explain why friendship might be possible with them but not with, say, a cactus). Likewise, Crary states that knowing whether an animal is of a kind “that characteristically [possesses] psychological qualities” or whether she is of a kind “so primitive that [she lacks] any characteristic psychological qualities” is of importance for determining the *sort* of respect she merits [1] (p. 48). The importance of not leaving aside all biological facts is especially salient in cases of uncertainty. Caring for a dog may not require much study of her biological facts, because we are sufficiently acquainted with this species, but insight into other, unfamiliar species, such as octopuses, may be crucial in giving them the treatment they are owed. The point of Crary and Diamond’s insight is not that biological facts must be completely left aside, but rather, that they are not *all* one needs to know. Biological facts, without the aid and interpretation in light of practices, can neither ground moral action nor moral theory. Therefore, while some naturalism is unavoidable, and indeed desirable, we should aim for an ethics that is *empirically informed*, and steer clear of misguided attempts to *reduce* moral life to what can be scientifically described.
(3) Reductionism: Lastly, traditional MI is *reductionist* because it tries to reduce our moral lives to a theory that postulates as few principles as possible and is even monistic in some cases. The reason why so many scholars are drawn to utilitarianism despite its highly counter-intuitive implications is because utilitarianism is the ethical theory that requires assuming the least. All one needs is to postulate a hedonistic account of the good (which, being a monistic theory, postulates the minimum in terms of values) and an aggregation principle. It is seen as a virtue of this theory over all others that it requires us to assume so little. However, this is only a virtue if one assumes that moral theory should strive to be as close as possible to mathematical theory and not to our moral life. It is not at all clear why this should be so. Instead, one could turn the logic of this argument on its head and argue that precisely *because* utilitarianism reduces our whole moral lives to the simple claim “pain is bad; pleasure is good; the least pain and the more pleasure there is, the better” that it should be rejected. Along these lines, Midgley argued that any moral philosophy that tries to reduce our moral lives to a “simple formula” can in the end only be considered simplistic and deceitful [49] (p. 30).
  This mismatch with our lived morality is precisely the reason why the Wittgensteinians criticise traditional MI for being reductionist and see the plurality of moral life as the key to ethics. Although theories developed in the traditional MI framework may have the virtue of logical consistency, they leave aside all the richness and complexity of our moral lives. This is all the more problematic if one takes into account, as argued in the previous point, that it is precisely from our moral lives and established practices that traditional MI ultimately derives its conclusions and normative power. The case against speciesism, for instance, would be impossible to construct were it not for the case against slavery, racism, and sexism that is used as an analogy.^14^ Moreover, by being so reductionist, these theories ultimately become uncompelling precisely because they are simply unable to account for *all* of our deeply held moral beliefs, which we might not want to give up *precisely* for moral reasons. The reasons we would employ care in our dealings with a disabled person, our baby, our neighbour, our grandma or a dead body may all be completely different. An over-emphasis on principles, logical consistency and the least amount of values possible will necessarily leave some of these reasons aside, which will make the resulting moral theory paradoxically incapable of accounting for all our well-established moral practices. In this sense, while the orthodoxy has on its side the virtues of logical coherence and the capacity to simplify the messiness of our moral lives, the Wittgensteinian approach has a potential for higher explanatory power, insofar as leaving aside reductionism and embracing pluralism could allow us to make sense of a higher proportion of our moral beliefs.

## 5. An Alternative to the Orthodoxy?

In the previous sections, we predominantly deal only with the negative side of the Wittgensteinian approach to animal ethics. Apart from delineating the limitations of their critique of MI, we also identify some of its promising aspects. However, to consider whether the Wittgensteinian approach can constitute a solid alternative to the orthodoxy, there is a critical point that needs to be dealt with. This is the idea of giving priority to practice and paradigms instead of theory and principles, which has been questioned not only in animal ethics (e.g., [32]), but in applied ethics in general (e.g., [50] (pp. 34–77); [51]; [52] (pp. 384–413)) and moral epistemology in particular (e.g., [53,54,55]. The key point boils down to the question of whether moral judgments can be substantiated with reference to established practices and whether this would keep ethical argumentation caught in what is morally accepted presently (thus “[turning] ethics into sociology” [32] (p. 37)) or, even worse, run the risk of reproducing a given, morally illegitimate practice. Could we ever have come to the idea that the burning of “witches”, slavery, racism, sexism, etc. are morally wrong if the only resource of ethical critique was practice? To answer this crucial point, we would like to put forward three arguments in support of the idea that giving priority to practice in ethical thought does not automatically mean losing the potential to criticise practices.

First, the worry that assuming the priority of practices implies that these cannot be criticised seems to rest on the presupposition that theory and principles (as opposed to practice and paradigms) are the only sources of moral criticism. However, this rests on shaky grounds, because, if true, it would imply that we need the discipline of moral philosophy to be able to question a given practice. However, this is clearly not the case, since adults who have no familiarity with moral philosophy, as well as children, are perfectly capable of criticising and opposing the practices of others. As Kuusela states:

[L]eading a human life involves [a pre-theoretical] understanding of a distinction between good and bad. We do not need the discipline of ethics or moralists to teach us that there is such a distinction, but it already informs our lives.

[56] (p. 39)

Moreover, the opponent of this view would have to claim that, in pre-philosophical societies, moral problems would not exist and people would have no chance of moral change and progress. We take it as a given that this would be an absurd position to take.

Second, moral problems first emerge in practice and not as a result of theory. For instance, take the practical example of a veterinarian being asked to euthanise a healthy puppy because her coat colour does not match with breeding goals [57]. Hartnack et al. [57] showed that veterinarians in Austria hold a very clear attitude towards such cases and experience moral stress if pressed to euthanise the puppy purely on the basis of the owner’s wishes. Conflicts between the owners’ wishes and their responsibility for the animal’s life and welfare surface as moral challenges without reference to moral theory or principles. This would not be possible if our moral life and practices were immune to doubt and criticism without the assistance of moral theory and/or principles. If moral theory and/or principles were the only source of moral critique, moral critique (whether justified or unjustified) and the clashing of moral beliefs would be a rare phenomenon, which in fact is not the case. Multiple ethical theories have dealt with answering the question of how moral problems emerge and an important answer has been found in our pre-reflexive practices. For instance, Ross’s idea of conflicts between prima facie duties [58], Hare’s conflicts between pre-reflexive intuitions [59] or Dewey’s mention of the ill-functioning of habits ([60] (p. 192); [61] (p. 164f.)) all refer to practical problems that trigger moral deliberation (cf. [62] (pp. 141–151)).^15^

The main point to make is that critiques of practice-oriented accounts tend to see practices as non-dynamic; as fields of action where actors share an identical set of moral beliefs and understandings that do not collide. If this were true, practice-based accounts would indeed have a severe problem, since the critical potential of ethical thought would be lost and existing practices and their moral infrastructure would be reproduced and stabilised. Indeed, the core question of moral philosophy—“What should I do?”—would be obsolete, since there would be no need for orientation and moral guidance, such as in a Kantian kingdom of ends. However, this idea of practices is obviously misguided. Instead, moral problems emerge in practice *before* ethical theories are applied. Such situations of doubt and loss of orientation generate in people a need to *think* in order to regain orientation and guidance. In this regard, ethical theory and principles can be of great support, which brings us to the third point, that moral theory and moral principles can be integrated in practice-oriented accounts.

Third, not all practice-oriented theories oppose moral theory and principles. Under a more charitable reading of the Wittgensteinian account than defended, for instance by Aaltola [32], practices and theory are not “polar opposites” [32] (p. 31). Instead, it could be argued that an instrumentalist account of moral principles and theory, such as the one developed by Dewey, is compatible with a Wittgensteinian approach to morality. Dewey writes:

[T]he object of moral principles is to supply standpoints and methods which will enable the individual to make for himself an analysis of the elements of good and evil in the particular situation in which he finds himself. […] A moral principle, then, is not a command to act or forbear acting in a given way: it is a tool for analysing a special situation.

[61] (p. 280, emphasis in the original)

Such an instrumentalist account would allow for theory and principles to still play a major role in ethics despite the fact that practice is put first. They have the function of guiding our practical thinking, and they can do so in a significant way, but they serve as instruments, not as ends. One can use the metaphor of a walking stick to think of moral theory and principles in this context: whereas a walking stick will provide support to walk better, it cannot make someone walk. One does not walk *because* one has a walking stick. Instead, one first needs the capacity to walk, and only then can one use the walking stick to get from A to B better. Moral theory and principles can be seen as tools to reason more thoroughly, in more detail and with higher degrees of sophistication, but they are neither primary to moral practices nor should they be viewed as ends in themselves.

With regard to animal ethics, moral theory and principles can make morally significant aspects visible. However, to use such tools and search for what is of moral significance in a given situation, the experience that there is something wrong and the practical need for orientation is prior. To give a second illustration from the veterinary context: veterinarians are not only legally responsible to care for and cure animals, but also have to cull healthy animals in the case of, e.g., zoonotic outbreaks. It is not surprising that these culling practices are regularly criticised. Moral theory and principles can help to systematically deal with the problems, and determine whether culling is unjustified harm or not; however, the experienced need for guidance comes first. Without such an experience, it is rather unlikely that practical reasoning will ever be applied, be it in real life or philosophical seminars. However, practices on their own, without the aid of ethical deliberation, will never manage to deal with these real-life problems in sufficient depth or with sufficient rigour. In this respect, the Wittgensteinian approach to animal ethics should not, we believe, strictly speaking be construed as an *alternative* to the orthodoxy. Adopting this approach would not imply relinquishing the orthodoxy altogether, but rather embracing moral theory as a tool to help us think better, a walking stick to help us along the way.

## 6. Conclusions

There are significant merits to the Wittgensteinian critique of MI. However, these merits pertain to the critique of the rationalism, naturalism and reductionism with which traditional forms of MI have been characterised. As we try to show, attempting to leave all attention to the individual aside in ethics is not a feasible option, and Crary and Diamond themselves are to a certain extent individualistic in their moral thinking. While their critique of the *individualism* in MI ultimately fails, we believe and indeed argue that there is much of value in their call for an ethics that is more *human*; an ethics that fully embraces the capacities we are endowed with and one that pays heed to the richness and complexity that characterises our moral lives. In addition, we argue that giving priority to practices does not mean that all normativity would be lost or that injustices must be perpetuated. Instead, we show that moral problems predate theory and that practices can be criticised in the absence of moral theory, but also that highlighting the importance of practices does not imply leaving moral theory aside. Rather than being an alternative to the orthodoxy in animal ethics, the Wittgensteinian approach can be seen as one that compliments, but also gives its proper place to the orthodoxy.

## Footnotes

1 Another prominent author who has applied Wittgenstein’s ideas to animal ethics is Raimond Gaita [3]. However, because we are interested specifically in the critique of MI developed by Crary in close connection with Diamond’s ideas, and because we consider Gaita to defend much more of an anthropocentric position than Crary and Diamond, we will be leaving his views aside. Consequently, we use the term ‘Wittgensteinian authors’ or ‘Wittgensteinians’ as a shorthand for Crary and Diamond.

2 In fact, only one of us (HG) self-identifies as a Wittgensteinian, and this paper is the result of an attempt to bridge the gap between Wittgensteinian and orthodox approaches to animal ethics.

3 Although a lively debate about Wittgenstein and moral philosophy has emerged in the last couple of years (e.g., Gleeson & Taylor [14]; De Mesel & Kuusela [15]; De Mesel [16]; Agam-Segal & Dain [17]; De Mesel & Thompson [13]), the animal issue has for the most part been ignored. Three positive exceptions are Richter [18], Crary [19], and Taylor [20].

4 For a detailed discussion of the argument from species overlap and the surrounding debates, see Dombrowski [22], Horta [21].

5 It should be noted that humans may be no different from other animals in not wanting to eat their conspecifics. This makes sense biologically, since diseases are more likely to be transmitted within rather than between species, which makes consumption of other species less risky.

6 An argument on how to integrate “objectivity” in Wittgensteinian ethics has been put forward by DeMesel [16] (p. 91–110).

7 We write ‘change’ instead of merely ‘difference’ because, despite departing from Rachels’ definition, in the bulk of the text Crary uses the term ‘capacities’ instead of ‘characteristics.’ She does not explain the reason behind this choice of terminology [1] (pp. 20).

8 Delon himself alternates between these two terms, but he doesn’t use ‘moral intrinsicalism’ as a synonym for MI, but rather as a way of labelling the foundational assumption of MI, which, following McMahan, he takes to be that “only intrinsic properties can be status-conferring and give rise to agent-neutral moral reasons” [8] (p. 357; cited in [26] p. 33).

9 One could argue that by making MI so broad as to include relation-, capacity-, and (as we shall shortly see) group-based reasons, we risk turning it into a principle that is of very limited use in moral theory, since it doesn’t narrow down which characteristics are actually relevant in moral theorising. But this is precisely the point we want to make: MI is an axiological- and normatively-neutral principle, and as such very little follows from it. Moreover, its broad nature reflects the point we aim to make in this section: MI is ultimately inescapable when one is engaged in ethics, and so it is misguided to direct one’s argumentative efforts against it.

10 Diamond’s attempt to defend the significance of being human has been criticised for reproducing the “anthropocentric dualism” [32]. However, we believe that under a more charitable reading it does not amount to a defence of anthropocentrism, for Diamond (like Crary) does not claim that it is the *only* morally relevant characteristic. In Diamond’s words: “To treat the notion of *human beings* as important in moral thought, as I have, is not to treat animals as outside the boundaries of moral concern, because it is not any kind of attempt to determine the limits of moral concern” [33] (p. 59, her emphasis). Nevertheless, although Diamond’s account is open to practical change, it runs the risk of preserving and stabilizing current practices. Accordingly, Aaltola criticizes Diamond’s account (and casuistry) on the basis that “[…] the nature of normativity is lost if it is reduced to description” [32] (p. 37). We acknowledge this problem, and deal with it in more detail in Section 5.

11 Crary and Diamond criticise MI for their excessive focus on *capacities* as criterion of moral consideration, and for the reductionism that comes with this. We will discuss their critique of the reductionism in MI in the following section. The orthodoxy’s excessive focus on capacities has also been taken to amount to a form of anthropocentrism, since the capacities that are usually taken as criteria for moral consideration are capacities that are important *for humans*. For a more extensive debate on the implicit anthropocentrism in animal rights theory and animal ethics in general, see Wolfe [34]; Grimm [35]; Hadley [36].

12 The way in which defenders of hedonism respond to this sort of criticism is by saying that objective harms are morally relevant because they typically impact subjective experiences. Thus, they might respond that the reason why it is wrong to deface or mutilate a corpse is because it would be distressing to the friends or family of the dead individual, who wouldn’t like to see her memory tarnished. The problem with this sort of response is two-fold: first, it doesn’t explain the intuition that the dead individual *herself* is being wronged by this treatment, and second, it makes the existence of harm entirely contingent on the presence of someone witnessing the deed.

13 Diamond seems to be aware of this, for she writes that the difficulty that the orthodox approach to animal ethics finds in justifying our treatment of dead and disabled humans “is a matter of its hardness for any approach to morality deriving much from utilitarianism—deriving much, that is, from a utilitarian conception of what makes something a possible object of moral concern” [12] (p. 469).

14 The fact that sexism and racism are used as an analogy to explain what is wrong with speciesism does not mean that this analogy is entirely valid. Diamond, for instance, criticised it by claiming that the relationship between these different forms of domination is not as “simple and straightforward” as traditional moral individualists would have it [12] (pp. 466–467). The point is not that this analogy is a valid one, but rather that it’s impossible to ground a moral theory without reference to our practices.

15 Naturally, also moral theory and principles can trigger moral deliberation, however, the point being made here is not that practices are the *only* source of moral deliberation, but that problems that emerge in practice are a common trigger for ethical deliberation.
